# Factor structure of mood over time frames and circumstances of measurement: Two studies on the Profile of Mood States questionnaire

**DOI:** 10.1371/journal.pone.0205892

**Published:** 2018-10-15

**Authors:** Elena Andrade, Dolores Rodríguez

**Affiliations:** 1 Department of Social Psychology and Methodology, Faculty of Psychology, Universidade de Santiago de Compostela, Santiago de Compostela, A Coruña, Spain; 2 Department of Clinical Psychology and Psychobiology, Faculty of Psychology, Universidade de Santiago de Compostela, Santiago de Compostela, A Coruña, Spain; University of Zaragoza, SPAIN

## Abstract

This paper presents the results of two studies on the invariance of the Profile of Mood States questionnaire across response time frames and circumstances of administration. We applied Spanish versions of the instrument to gather data from 1146 athletes. In the first study (N = 700), we tested the factor structure of the questionnaire in training sessions by using two different time frames: ‘right now’ (n = 350) and ‘past week’ (n = 350). In the second study (N = 446), we compared the factor structure of the questionnaire with data collected using the instruction ‘right now’ at two different circumstances: ‘training’ (n = 223) and ‘competition’ (n = 223). Data analysis was similar in both studies. We conducted multi-group confirmatory factor analyses and applied the scaled difference chi-square statistic to examine whether discrepancies in successive constrained models were significant. We observed configural equivalence between the two time frames. Furthermore, we observed metric equivalence but not scalar invariance between the different circumstances of measurement. The findings highlight the need for studies of equivalence before using a single self-report with more than one set of instructions, or under diverse circumstances. Invariance of mood scores should be examined and taken into account when interpreting individual and group mood state assessments.

## Introduction

Self-report measures of mood are predominant in the psychology domain, and have been extensively used for exploring subjective mood states in sports people. Compared to other subjective experiences like emotions, moods are described as less intense but more enduring, diffuse, and often not attributable to a clear cause [1]. Existing mood measures have a multidimensional character, and include scales aiming to cover both positive and negative components [2]. In these scales individuals use adjectives to describe their current mood, which is profiled in terms of multiple specific states.

One of the most popular mood states measures is the Profile of Mood States (POMS). The Standard English version [3] comprises 65 items rated on a five-point response format. Participants can select a number from 0 (*not at all*) to 4 (*extremely*). Fifty-eight of the original items yield scores on six factors: Tension, Depression (Depressed mood), Anger, Vigour, Fatigue, and Confusion. Another seven items represent Friendliness.

Friendliness was difficult to replicate in some validation studies conducted by the authors of the POMS [3]. This is the reason why researchers have systematically excluded the items representing the seventh mood state, which has fostered the criticism of the ineffectiveness of the POMS for dealing with positive, pleasant feelings [4]. Recent editions of the original test, however, assert that Friendliness can be considered as a separate indicator of interpersonal functioning [5].

The literature on the POMS documents its use for measuring mood states in diverse research and applied fields of psychology, and reflects the great popularity of this instrument in the field of sport [6]. In the POMS athletes show a characteristic pattern of scores, widely known as the iceberg profile, a model that may help predict sports performance [1, 2]. Lane and Terry [1] developed a conceptual schema of the effect of mood states on performance based on the interactions among the POMS’ factor scores. Their theoretical model emphasised the relationships of depressed mood state with other unpleasant states, particularly tension and anger.

The POMS is available for use on both adults and adolescents, and the questionnaire has been translated into more than fifteen languages. From a methodological perspective, its internal structure seems relatively established, with the exception of the Confusion factor [7–9], which was regarded as a cognitive state [2]. Moreover, despite the limited range of pleasant mood states covered, for the aforementioned reasons, few adaptations retained the component of Friendliness [4].

Concerning the instructions, the requests ‘How have you felt during the past week including today?’ and ‘How do you feel right now?’ are the most commonly used in the POMS.

McNair et al. [3] chose the reference to the past week since they considered this was a long enough period to capture people’s typical and persistent emotional reactions to daily life events, yet short enough to assess the acute effects of a treatment. Nonetheless, they also indicated the possibility of using other instructions, depending on the purpose of the study. In one factor analysis, McNair and co-workers replicated the original structure by using the ‘right now’ instruction with a sample of college students [3].

Some researchers have examined the consequences of switching reference times in mood assessment. According to Watson [10], the structure of positive and negative affect factors emerged regardless of the time frame rated.

Regretfully, most of the subsequent inquiries focused on observed factor means, and were not preceded by an analysis of measurement invariance. The results were, nevertheless, of interest, and served to justify the selection of the type of instructions. As stated by Winkielman, Knauper, and Schwarz [11], people can adjust their reference periods to rate their mood states. Thus, the scope of the reference period will affect an individual’s perception of intensity, as well as of seriousness and frequency of the episode under evaluation [11, 12].

This impact on mood state ratings was identified in the Anglo-Saxon context both in adults and in adolescents. For instance, Terry, Stevens, and Lane [13] found that the scores obtained in assessments of 135 school children based on the ‘past week’ were higher than the average scores obtained in multiple ‘right now’ assessments. Recall of mood appeared to be influenced by mood at the time of recall and possible significant events. So Terry et al. [13] suggested that the ‘right now’ response time frame should be the method of choice.

As a complementary argument supporting the instruction ‘right now’, some authors affirm that this temporal reference of ‘right now’ is more sensitive to demands of the situation. In the sports setting, it is therefore believed more useful for assessing the relationship between mood states and subsequent performance [14, 15].

Adaptation of the type of instructions to the research objective seems reasonable. However, the impact of changes in reference period on psychometric properties related to the use of the instrument must be tested. Thus, the first step should be to demonstrate the degree of invariance of the measurement model across time frames. Otherwise, we could judge a person’s score or make comparisons among group means which are not supported by the underlying structure.

Previous research [10, 11, 13, 15] has identified additional factors (e.g., diverse mood state descriptors, response formats, or circumstances of assessment) that could affect the understanding and comparability of the mood state responses. Of all the potential modulators, one variable stands out: the circumstances of mood state assessment, i.e., the conditions of time and place under which the mood state response is registered. In this respect, the POMS questionnaire has been administered before and after competition, and outside the competition context [15, 16].

Terry and Lane [15] speculated that mood states would differ significantly depending on the conditions in which the athletes were assessed. Terry, Lane and Fogarty [16] examined whether the structure of the POMS could be reproduced in disparate samples of adolescent and adults in two different situations: before competition and in the classroom setting. Although in the expected direction, the results of multi-group confirmatory factor analyses were adequate only when factor structure was constrained to be equal across the different samples. The fit statistics were considered marginal when factor loadings and factor covariances were also restricted to be equal. To our knowledge there were no further probes of invariance of the English POMS over different assessment circumstances.

Both the type of instruction and the circumstances of administration of the test are important measurement elements.

As regards the Spanish context, two versions of the POMS have been published recently; these are adaptations of the questionnaire for use with adult athletes and the general population [17] and with adolescent athletes [18]. However, two different sets of instructions have been applied, and the questionnaire has been administered during training and just prior to competition [19]. In the absence of probes of invariance, it will be difficult to establish whether mood state deviations between instruction groups or over time are due to real differences or to changes in the construct measure [20].

Our aim is to tackle the problem by analysing these two important psychometric aspects of the use of the POMS: the time-frame instructions, and the circumstances of administration of the questionnaire.

We report the results of two studies in which the POMS was administered to Spanish athletes. In the first study, we compared the factor structure of the questionnaire with data obtained using two different sets of instructions: ‘right now’ and ‘past week’, during training. In the second study, we compared the factor structure of the questionnaire with data obtained using the instruction ‘right now’ at two different circumstances: ‘training’ and ‘competition’.

## Method

### Participants

The data were originally recorded for the purposes of improving the instruments. Analysis of the time frame was performed by comparing the responses of two groups of Spanish adult athletes. The sample comprised 700 individuals: 428 (61.1%) men and 272 (38.9%) women, of mean age 22.51 years (SD = 5.53, range = 16–53).

Data on years of education, type of sport and competitive level were not always available, because they were not part of the information collected in those cases. However, the available information shows the proportions of participants who had completed or were undertaking the following types of education: university courses (65.15%), post-compulsory schooling (13.18%), vocational studies (11.86%) and compulsory education (9.81%). The percentage of participants involved in different types of sport was as follows: soccer (37.4%), basketball (9.7%), rowing (26.6%), chess (8.6%), athletics (4.3%), indoor soccer (3.7%) and fencing (3.7%). The remaining 6% of subjects participated in less well represented sports such as hockey, tennis, swimming, water polo, cycling and handball. Of the sample, 68.6% competed at regional level and 31.4% at national level.

A small number of participants were still in adolescence. It might be appropriate to clarify that, adopting the same argument given by the authors of the instrument that was used [17], those athletes were kept in the study, since they trained and competed within the top category teams, and they did not show extreme values in the variables of interest.

The group who received the instruction ‘right now’ comprised 350 participants: 182 (52%) men and 168 (48%) women. Their mean age was 24.04 years (SD = 7.25, range = 16–53).

The group who received the instruction ‘past week’ comprised 350 participants: 246 (70.3%) men and 104 (29.7%) women. Their mean age was 21.03 years (SD = 2.20, range = 17–33).

We estimated the differences between the groups in terms of sex and age. This differences were significant, with low to moderate effect sizes: for sex, X^2^(1) = 24.63, p < .01, η^2^ = .035; for age, F(1, 687) = 55.36, p < .01, η^2^ = .075.

Analysis of the circumstances of measurement was performed with data from two groups of Spanish adolescent athletes who received the same instruction: ‘right now’. The groups comprised a total of 446 participants: 344 (77.1%) men and 102 (22.9%) women, aged between 13 and 18 years (M = 15.13, SD = 1.20). The distribution of participants per sport was as follows: basketball (50.67%), soccer (14.35%), indoor soccer (14.80%), volleyball (9.19%) and chess (4.71%). The remaining 6.28% participated in handball, water polo, fencing and athletics. Of the sample, 48.5% competed in regional league divisions, and 51.5% competed in local categories.

The first group comprised 223 athletes measured during training. One hundred and seven (71.7%) were men and 63 (28.3%) were women. Their mean age was 14.86 years (SD = 1.20, range = 13–18).

The second group included 223 athletes measured just before competition. One hundred and eighty-four (82.5%) were men and 39 (17.5%) women. Their mean age was 15.39 years (SD = 1.14, range = 13–18).

The differences between the groups is terms of sex and age were significant, with low effect sizes: for sex, X^2^(1) = 7.32, p < .01, η^2^ = .02; for age, F(1, 440) = 22.11, p < .01, η^2^ = .05.

### Instruments

We used a Spanish form of the POMS questionnaire designed for adult athletes to assess the type of instruction [17]. This comprised 30 evenly distributed items (five per factor) in six first-order factors: tension, depression, anger, vigour, fatigue and friendliness. Examples of items included in the questionnaire were the adjectives *angry*, *energetic* or *sad*.

The response format consisted of five ordered categories scored between 0 and 4. The psychometric properties for this measurement model in athletes were documented by Andrade et al. [17], who reported satisfactory values of reliability (with consistency estimations between .79 and .87) and the following goodness-of-fit statistics: X^2^(390, N = 400) = 803.12, p < .01, CFI = .97, NNFI = .97, RMSEA = .051, and SRMR = .057.

We used a Spanish version of the POMS questionnaire designed for adolescent athletes to compare the circumstances of measurement [18]. This comprised 29 items, four for fatigue, and five each for the factors tension, depression, anger, vigour and friendliness.

The items were rated in five ordered categories that received scores between 0 and 4. Andrade et al. [18] reported adequate values on the internal structure of the test, which was subjected to confirmatory factor analysis (fit values of X^2^(361, N = 320) = 684.38, p < .001, CFI = .96, NNFI = .96, RMSEA = .053, and SRMR = .061.), and on its reliability, estimated by the Cronbach’s alpha statistic (with values between .77 and .87).

### Procedure

The research involved the collective, anonymous administration of tests. It did not require the approval of the University bioethics committee, which would be necessary in case of ‘personal character data subjected to a reserved treatment, which affected the rights and liberty of people, the interests linked to preservation of environment or of other legally protected goods.’ The project observed the ethical principles regarding participants [21]. People responsible for the teams and participants gave verbal consent and were informed about: (a) the objective of the study, expected duration and procedures; (b) their right to decline to participate and to withdraw from the research once participation had begun (that is, the participation in the study was voluntary); (c) the protection of confidentiality; and (d) the opportunity to ask questions and receive answers.

The POMS questionnaire was administered to both groups of adult athletes just before a usual training session. Three sport psychologists gathered the data in situ. The questionnaire was administered in a similar way in both groups by following a standardized protocol. However, the type of instruction was different. In one group, participants were asked to respond according to how they felt at that precise moment (‘right now’ instruction). Participants in the other group were asked to respond according to how they had felt during the past week, including the day of the test (‘past week’ instruction).

Another three psychologists administered the POMS questionnaire to the sample of adolescent athletes. The type of instruction was identical for the two groups, but the circumstances of administration of the questionnaire were different. The questionnaire was given to the first group immediately before a training session (‘training’ group); the same form of the questionnaire was given to the second group within the 30 minutes before a competition (‘competition’ group).

### Data analysis

The results are presented per question under study, although the analytical sequence was similar. Firstly, we tested the hypothesised measurement model in each group. The estimations were performed with LISREL 8.80, and satisfactory single-sample model-fit was represented above all by the conventional values of CFI and NNFI > .95, and RMSEA < .06. Secondly, we conducted multi-group confirmatory factor analyses by imposing successive restrictions on the model. We planned to perform the following tests: configural invariance (equivalent pattern of fixed and free factor loadings across all groups), metric invariance (equality of factor loadings), scalar invariance (equality of intercepts), latent means invariance, and invariance of relations among latent factors. We applied the scaled difference chi-square statistic to examine whether discrepancies in the successive constrained models were significant [22, 23].

## Results

### Study on the time frames

The data used to compare time frames did not fulfil the assumption of multivariate normality, with relative kurtosis values of 1.216 in the ‘right now’ group and 1.120 in the ‘past week’ group. The robust maximum likelihood method was therefore chosen as the estimation method [24].

The factor structure included 30 items, five for each of the postulated first-order factors. The model was over-identified with 390 degrees of freedom.

In the ‘right now’ group, the results for the goodness-of-fit indices were as follows: SBX^2^(390, N = 350) = 607.65, p < .001, CFI = .98, NNFI = .98, RMSEA = .040 (CI 90% [.034, .046]) and SRMR = .058. The range of the standardized factor loadings was between .43 and .88, and between .044 and .076 for typical errors. The inter-factor correlations ranged from .09 (relationship among vigour and tension) to .82 (relationship between anger and depression).

In the ‘past week’ group, the results for the most usual indices were as follows: SBX^2^(390, N = 350) = 734.66, p < .001, CFI = .97, NNFI = .97, RMSEA = .050 (CI 90% [.045, .056]), and SRMR = .074. Standardized factor loadings ranged between .52 and .91, with typical errors between .040 and .075. With respect to inter-factor correlations, the values ranged between .04 (for friendliness and fatigue) and .64 (for anger and tension).

As the posited structure was acceptable in both groups, we conducted a simultaneous analysis of configural equivalence. Both the number of factors and the factor-indicator correspondence are the same. This solution (see Table 1) will serve as baseline for posterior invariance proofs.

**Table 1:** Results from single and multi-group confirmatory factor analyses with respect to the time frames.

Type of analysis	SB X^2^	NT X^2^	ML X^2^	*df*	NT X^2^ diff ML X^2^ diff	*Δdf*	CFI	NNFI	RMSEA (90% CI)
Single group									
Right now (*n* = 350)	607.65	738.78	757.16	390			.98	.98	.040 (.034, .046)
Last week (*n* = 350)	734.66	834.61	859.50	390			.97	.97	.050 (.045, .056)
Measurement Invariance									
Configuration	1338.00	1573.40	1616.66	780			.98	.97	.045 (.041, .049)
Metric invariance	1437.76	1691.66	1730.08	810	100.22^a^	30	.97	.97	.047 (.043, .051)
					96.12^a^				

SB X^2^, Satorra and Bentler Chi-Square; NT, Normal Theory Weighted Least Squares Statistic; ML, ordinary Maximum Likelihood Statistic (Minimum Fit Function Value); CFI, Comparative Fit Index; NNFI, Non-Normed Fit Index; RMSEA, Root Mean Square Error of Approximation; SRMR, Standardized Root Mean Square Residual.

^a^ *p* < .05

We also tested the more restrictive model of metric equivalence. We used the scaled difference in chi-squares to compare both models. The difference was significant, which prevents us from inferring weak invariance.

By comparing the non-standardized factor loadings for both groups, together with modification indices, we identified the most problematic items (e.g. the item *weak*, representing the factor fatigue). We decided to proceed in a context of partial invariance. However, any plausible nested model implied a significant decrease in goodness-of-fit.

### Study of the circumstances of measurement

The CFA was conducted in the groups that were assessed at two distinct circumstances with the method of robust maximum likelihood, because the data distribution was not multivariate normal. The values of relative multivariate kurtosis were 1.223 in the ‘training’ group and 1.221 in the ‘competition’ group.

The measurement model, with 29 items and six first-order factors, was also over-identified, with 362 degrees of freedom.

In the ‘training’ group, the results for the goodness-of-fit indices were as follows: SBX^2^(362, N = 223) = 535.47, p < .001, CFI = .97, NNFI = .97, RMSEA = .046 (IC 90% [.038, .055]) and SRMR = .070. Standardized factor loadings ranged between .34 and .90, with typical errors between .062 and .091. The correlations between latent factors adopted absolute values between .10 (for friendliness and tension) and .63 (for anger and tension).

In the ‘competition’ group, the results for the fit indices were as follows: SBX^2^(362, N = 223) = 558.02, p < .001, CFI = .95, NNFI = .94, RMSEA = .049 (IC 90% [.041, .057]) and SRMR = .080. The factor loadings ranged between .13 (for carefree) and .86 (tired), with typical errors between .063 and .096. The correlations between latent factors yielded values between .03 (for friendliness and vigour with tension) and .51 (for friendliness and vigour).

The simultaneous analysis of configural equivalence (Table 2) showed an acceptable data fit. The solution of equality of factor loadings did not produce a significant decrease in the goodness-of-fit, which allows us to assume metric equivalence. Respondents across groups attribute the same meaning to the latent construct under study.

**Table 2:** Results from single and multi-group confirmatory factor analyses with respect to the circumstances of measurement.

Type of analysis	SB X^2^	NT X^2^	ML X^2^	*df*	NT X^2^ diff ML X^2^ diff	*Δdf*	CFI	NNFI	RMSEA (90% CI)
Single group									
Training (*n* = 223)	535.47	679.20	657.09	362			.97	.97	.046 (.038, .055)
Competition (*n* = 223)	558.02	703.14	716.69	362			.95	.94	.049 (.041, .057)
Measurement Invariance									
Configuration	1093.41	1382.34	1373.78	724			.96	.96	.048 (.042, .054)
Metric invariance	1138.22	1452.21	1434.25	753	39.70^a^	29	.96	.96	.048 (.042, .054)
					34.36^a^				
Scalar invariance	1195.10	1503.34	1483.16	776	83.82^b^	23	.95	.95	.049 (.044, .055)
					80.18^b^				

SB X^2^, Satorra and Bentler Chi-Square; NT, Normal Theory Weighted Least Squares Statistic; ML, ordinary Maximum Likelihood Statistic (Minimum Fit Function Value); CFI, Comparative Fit Index; NNFI, Non-Normed Fit Index; RMSEA, Root Mean Square Error of Approximation; SRMR, Standardized Root Mean Square Residual.

^a^ *p* > .05.

However, the more restrictive model of scalar equivalence indicated a significant increase in the difference between chi-squares. Therefore, scalar invariance was not observed with these samples.

Given that factor weights and the indicators intercepts are not invariable in both samples, the difference in the latent means between them cannot be interpreted.

## Discussion

The aim of this paper was to examine the invariance of the factor structure of the POMS questionnaire across the type of instruction and the circumstances in which the measure was taken. Both aspects are relevant for meaningful interpretation of scores in self-reports.

To assess mood states, researchers ask participants to rate the intensity or frequency with which they had experienced certain feelings during a period of time. However, in these situations the same word may represent a wide range of distinct experiences. If the meaning of the word is not specified by the researcher, participants might infer it from some characteristics of the measurement instrument. One such characteristic is the length of the reference period [11, 12].

From the results of previous studies with the POMS in Spanish [17, 18], it seems that the use of one or another type of instruction does not compromise the general psychometric quality of the measure. Nonetheless, this does not imply that the character of the scores and the information they provide to researchers and professionals is the same in all cases.

In our first analysis, we compared the concurrent (‘right now’ instruction) and retrospective (‘past week’ instruction) reports provided by the Spanish POMS. By following strict goodness-of-fit criteria, we found that there was configural equivalence over the two sets of instructions. However metric invariance was not endorsed. In other words, both the number of factors and the factor-indicator correspondence are the same, but the unstandardized factor loadings of each indicator are not equal across the compared groups.

From a methodological point of view, Terry et al. [16] cited among the possible causes of misfit some adjectives representing fatigue. Similarly, the item *weak* was one of the most problematic markers when testing metric invariance in our study.

If configural invariance is verified but metric invariance is not, one can address it by identifying and releasing problematic indicators if there are empirical and substantive reasons to do so [25]. We considered this possibility, but did not find a plausible model which statistically satisfied the criteria of goodness-of-fit. On the other hand, partial measurement invariance could be highly dependent on chance characteristics of the observed samples, and its consequences on reliability and estimation of latent parameters are yet to be determined [20].

As per our first examination, we must conclude that the respondents across groups did not attribute the same meaning to the latent construct under study. This outcome supports the need for separate normative values for observed scores gathered with each set of instructions.

From a conceptual perspective, a possible explanation for the lack of metric equivalence could be that the ‘past week’ reference is a measure of long-lasting mood states [26], while the ‘right now’ reference represents situationally-sensitive states [15, 27]. Although adherence to discrete state or trait concepts is an oversimplified approach to the measurement of mood state dimensions, it should be one of the considerations during decision on which time frame to choose.

Another aspect of great importance regarding de use of the POMS are the circumstances of administration of the questionnaire. Depending on the survey objectives, the situation would sometimes require proximity between the measure of mood states and the stressful event; at other times the researcher would be interested in assessing mood states during the training phase, to monitor mood states rather than for making decisions about immediate intervention. As for the results obtained in our second analysis, it appears that even in relatively small samples there is metric equivalence across the two measurement moments, training and competition. Thus, the constructs are manifested the same way in each group.

However, scalar invariance was not fulfilled, so we cannot assume that we would obtain the same observed score for an indicator at a given level of the latent factor. Groups cannot be compared on their scores on the latent variable.

We judge this finding congruent with personality psychology which conceives behaviour over time as consisting of density distributions of states [28]. Differences in behaviour are also best described as density distributions, and the individual differences that show regularity are those distributions of states rather than single scores or only the mean or another unique parameter of the distributions.

Definitely, new systematic investigation is needed to test hypotheses about the aspects which affect mood states measurement in general and about invariance relative to the application of the POMS questionnaire in particular. This paper represents a first approach, and is not intended to offer a final word on the matter.

It also holds some shortcomings that should receive attention in future research. One weak point of our work is that mood states scores based on the ‘past week’ constitute a retrospective measure, and factors potentially causing memory distortions could not be controlled [13].

Research would also benefit from designs in which the same group of participants assessed their mood states under the two different sets of instructions or over time. There are few antecedents in the literature, and their results are diverse. Galinha, Pereira, and Esteves [29] tested the temporal invariance of the PANAS (Positive and Negative Affect Schedule), using the structural equation modelling analysis, in a group of adults. As an interesting result, although they used its state version, the PANAS scale showed stability over a two-month interval. In contrast, Fried et al. [30] investigated unidimensionality and measurement invariance of four common depression rating scales (one self-report, three clinician-reports) in two large studies. For all instruments, neither unidimensionality nor measurement invariance were judged as acceptable.

Additionally, we could have considered less restrictive criteria to judge each multi-group confirmatory factor analysis model. As Cheung and Rensvold [31] pointed out, the outcome of the chi-square difference test could indicate the lack of measurement invariance even when the imposed equality constraints lead to minor decreases in model fit. One way to handle this is to compare the unstandardized parameter estimates across the solutions. Another is to review changes in alternative fit indices. For simplicity, only approximate fit indices were shown in the tables. Cheung and Rensvold [31] suggested that a change of just .01 in *CFI* values meant that the null hypothesis of invariance should not be rejected. Meade, Johnson, and Braddy [32] suggested that a change of .002 in *CFI* values implied just trivial deviations from perfect measurement invariance. Kang, McNeish, and Hancock [33] supported the increment in McDonald’s *NCI* as the index of choice.

The samples in our studies were not as large as those simulated in, for instance, Meade et al.’s paper. So we based our conclusions on the difference between chi-squares rather than on alternative fit indicators, thus adopting a more conservative attitude at this stage of research on the topic.

## Conclusion

This paper emphasizes the need for studies of equivalence before using a single self-report with more than one set of instructions, or under diverse circumstances. Particularly with respect to instructions, it shows that if we administer a mood states test with two distinct time frames (e.g. ‘right now’ and ‘past week’), we may be measuring the same conceptual dimensions, but with no guarantee of doing it with the same meaning.
